# Association of two novel systemic inflammatory biomarkers and frailty based on NHANES 2007–2018

**DOI:** 10.3389/fpubh.2024.1377408

**Published:** 2024-04-08

**Authors:** Huiling Zhang, Xinyu Liu, Xiaoling Wang, Ya Jiang

**Affiliations:** ^1^The First Clinical Medical College, Shandong University of Traditional Chinese Medicine, Jinan, China; ^2^Department of Cardiology, Linyi Traditional Chinese Medicine Hospital, Linyi, China

**Keywords:** frailty, SIRI, SII, systemic inflammatory biomarkers, NHANES

## Abstract

**Background:**

Frailty is a significant concern in the field of public health. However, currently, there is a lack of widely recognized and reliable biological markers for frailty. This study aims to investigate the association between systemic inflammatory biomarkers and frailty in the older adult population in the United States.

**Methods:**

This study employed data from the National Health and Nutrition Examination Survey (NHANES) spanning 2007 to 2018 and conducted a rigorous cross-sectional analysis. We constructed weighted logistic regression models to explore the correlation between the Systemic Immune-Inflammation Index (SII), Systemic Inflammatory Response Index (SIRI), and frailty in the population aged 40 to 80 years. Using restricted cubic spline (RCS), we successfully visualized the relationship between SII, SIRI, and frailty. Finally, we presented stratified analyses and interaction tests of covariates in a forest plot.

**Results:**

This study involved 11,234 participants, 45.95% male and 54.05% female, with an average age of 64.75 ± 0.13 years. After adjusting for relevant covariates, the weighted logistic regression model indicated an odds ratio (OR) and 95% confidence interval(CI) for the correlation between frailty and the natural logarithm (ln) transformed lnSII and lnSIRI as 1.38 (1.24–1.54) and 1.69 (1.53–1.88), respectively. Subsequently, we assessed different levels of lnSII and lnSIRI, finding consistent results. In the lnSII group model, the likelihood of frailty significantly increased in the fourth quartile (OR = 1.82, 95% CI: 1.55–2.12) compared to the second quartile. In the lnSIRI group model, the likelihood of frailty significantly increased in the third quartile (OR = 1.30, 95% CI: 1.10–1.53) and fourth quartile (OR = 2.29, 95% CI: 1.95–2.70) compared to the second quartile. The interaction results indicate that age and income-to-poverty ratio influence the association between lnSIRI and frailty. RCS demonstrated a nonlinear relationship between lnSII, lnSIRI, and frailty.

**Conclusion:**

The results of this cross-sectional study indicate a positive correlation between systemic inflammatory biomarkers (SII, SIRI) and frailty.

## 1 Introduction

Frailty, as a multifactorial syndrome, manifests a trend of increasing physiological system impairments with age and may significantly reduce survival rates at any age (1). A key characteristic of frailty is the gradual weakening of physiological systems and an exceptional sensitivity to various stresses and pressures in daily life (2). Frailty may trigger and exacerbate other health issues; therefore, prevention and slowing the progression of frailty are crucial. Frailty is determined by multiple indicators, including the Edmonton Frail Scale (3), the Geriatric Nutritional Risk Index (4), the Frailty Index (FI) (5), and the Fried phenotype, among others. Among these, FI performs well in distinguishing frailty states (6). An increased burden of inflammation also characterizes frailty (7). Systemic Immune-Inflammation Index (SII) (8) and Systemic Inflammatory Response Index (SIRI) (9) are novel markers linked with inflammatory conditions.

Blood inflammation markers are cost-effective and easily accessible biomarkers. Serving as indicators of both local immune response and systemic inflammation, SII is a robust and stable metric, integrating three types of inflammatory cells (lymphocytes, neutrophils, and platelets) (10–12). Numerous studies have indicated that the SII can predict the prognosis of patients with various cancers, acute ischemic stroke, heart failure, and acute kidney injury (13). SIRI, composed of lymphocytes, monocytes, and neutrophils, represents a more comprehensive indicator of chronic inflammation (14). Previous research suggests that SIRI is a potential marker for early diagnosis and prognosis monitoring in conditions such as stroke, inflammatory diseases, and cancer (15). Additionally, previous research has found an association between C-reactive protein and interleukin-6 with frailty (16).

Some studies have indicated an association between systemic inflammatory biomarkers and the risk of frailty (17). However, current research has limitations, including small sample sizes and a single definition of frailty. The universality and reliability of this association require validation and further confirmation through larger-scale studies. In this study, we conducted a rigorous analysis using a large sample from the National Health and Nutrition Examination Survey (NHANES) data spanning 2007 to 2018 to explore the correlation between SIRI, SII, and frailty. This research aims to gain deeper insights into the impact of systemic inflammatory biomarkers on frailty, ultimately providing more effective healthcare recommendations for individuals.

## 2 Methods

### 2.1 Study population

These data are available from the NHANES database (18). This database comprises a series of nationally representative surveys designed to assess U.S. citizens’ health and nutritional status (19). The database has received approval from the National Center for Health Statistics Ethics Review Board, and the participants have obtained written consent (20). We conducted data analysis on the most recent six NHANES survey cycles (2007–2018), encompassing 15,155 participants, followed by a series of exclusions.

1. Exclusion of participants with less than 80% of FI features (< 40 items) (n = 244);
2. Exclusion of participants with age < 40 years (*n* = 1,396);
3. Exclusion of individuals with insufficient baseline information (gender, race, education, marital status, income poverty ratio) (*n* = 1,402);
4. Exclusion of individuals with missing covariates (alcohol consumption, smoking) (*n* = 500);
5. Exclusion of individuals with missing values for SII and SIRI (*n* = 379).

**Figure 1:**
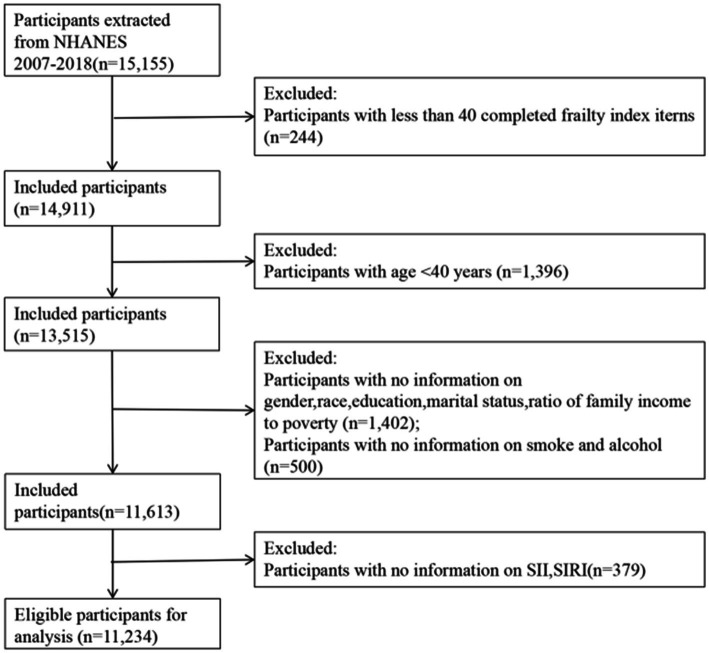
Flow chart of sample selection.

Ultimately, it includes 11,234 participants, as depicted in Figure 1.

### 2.2 Frailty

Following the approach proposed by Hakeem et al., we utilized the FI to assess the degree of frailty. This index comprises 49 variables spanning multiple systems, covering aspects such as cognition, dependency, depressive symptoms, comorbidities, general health status, hospital utilization, physical performance, body measurements, and laboratory test values (21–23). The eligibility survey required a completion rate of at least 80% (approximately 40 items) for the 49 frailty items. We assigned scores ranging from 0 to 1 based on the severity of defects (see Supplementary Table S1)^1^. The FI is the sum of defect scores obtained by participants divided by the potential total defect score. With a critical threshold for the FI set at 0.21, values greater than or equal to 0.21 are defined as frail, while values less than 0.21 are non-frail (24).

### 2.3 Systemic inflammatory biomarkers

An automated hematology analyzer will evaluate lymphocyte, neutrophil, platelet, and monocyte counts expressed as ×10^3^ cells/μL (25). We calculated two systemic inflammatory markers based on peripheral blood cell counts: SII and SIRI. The calculation formula for SII is platelet count × neutrophil/lymphocyte count (26). The calculation formula for SIRI is neutrophil count × monocyte count/lymphocyte count (27).

### 2.4 Covariates

Correlation logic and previously published literature guided the selection of covariates. We collected statistical data on basic participant information, including age, gender, race, education level, marital status, income poverty ratio, and statistics on alcohol and smoking habits. Specifically, age into two groups: <40 years and ≥ 40 years; gender into two groups: male and female; race into five groups: Mexican American, other Hispanic, non-Hispanic White, non-Hispanic Black, and other races, including multiple races; education level into three groups: less than high school, high school or general education diploma, and more than high school (28); marital status into three groups: married/living with a partner, never married, and separated/divorced/widowed (29); The income poverty ratio is categorized into three groups: low (≤1.3), moderate (1.3–3.5), and high (>3.5). It is calculated by dividing the family’s (or individual’s) income by the poverty threshold specific to the survey year. A lower income poverty ratio indicates lower income for the family (or individual). Based on the response to the question, “Have you smoked at least 100 cigarettes in your entire life?” participants were categorized as smokers or non-smokers. Based on responses to questions about drinking at least 12 alcoholic beverages in the past year and ever drinking any alcoholic beverage, participants were drinkers or non-drinkers (30).

### 2.5 Statistical analysis

In this study, statistical analyses followed the recommendations of the Centers for Disease Control and Prevention guidelines. We were considering the complexity and multi-stage sampling design of NHANES data collection and sampling weights in the statistical analysis. We compared the baseline characteristics between frail and non-frail individuals, as well as the baseline characteristics of different quartiles of the natural logarithm (ln) transformed SII (lnSII) and SIRI (lnSIRI). For normally distributed quantitative data, we used the t-test, and for qualitative data, we employed the χ^2^ test. Descriptive statistics presented continuous variables using mean ± standard deviation and categorical variables using percentages with 95% confidence intervals. Subsequently, weighted logistic regression models estimate the relationships between lnSII, lnSIRI, and frailty in three different models. Model 1 was a basic unadjusted model; Model 2 adjusted for age, gender, race, income poverty ratio, education level, and marital status; Model 3 included all variables in Model 2, plus smoking and drinking status. Furthermore, restricted cubic splines (RCS) were employed to detect potential non-linear relationships between lnSII, lnSIRI, and frailty. We performed Subgroup and interaction analyses for age, gender, education, marital status, income and poverty ratio, smoking, and drinking. All analyses use R software (V.4.3.2), Stata software (version 17), and SPSS software (version 27). Statistically significant: A significance level of *p* < 0.05 was considered.

## 3 Results

### 3.1 The baseline features of the participants

11,234 participants were included, with males comprising 45.95% and females 54.05%. Clinical characteristics of participants, stratified by frailty status, are presented in Table 1. The proportions of lnSII four quartiles among frail patients were 21.24, 21.80, 23.7, and 33.26%, respectively, while lnSIRI four quartiles were 20.08, 21.25, 25.01, and 33.67%, respectively. Frailty showed statistical significance (*p* < 0.05) concerning age, gender, race, education level, marital status, income poverty ratio, alcohol consumption, smoking status, lnSIRI, and lnSII. Compared to non-frail individuals, frail patients are often female, have lower educational levels, lower income poverty ratio, are less likely to be married or living with a partner, and are more likely to smoke. Additionally, they tend to have higher levels of lnSII and lnSIRI. The mean age of frail patients is 64.29 ± 0.22 years.

**Table 1:** Baseline characteristics of weighted sample by frailty and non-frailty groups.

	Total	Non-frailty	frailty	*p*-value
Sample size	1,1,234	6,690	4,544	
Age, Mean	64.75 ± 0.13	65.02 ± 0.16	64.29 ± 0.22	<0.001
lnSII, Mean	6.18 ± 0.01	6.14 ± 0.01	6.23 ± 0.01	<0.001
lnSIRI, Mean	0.17 ± 0.01	0.12 ± 0.01	0.25 ± 0.01	<0.001
Gender				<0.001
Men	45.95(44.63–47.27)	49.78(48.05–51.51)	39.27(37.32–41.26)	
Women	54.05(52.73–55.37)	50.22(48.49–51.95)	60.73(58.74–62.68)	
Race				<0.001
Mexican American	4.41(4.13–4.71)	3.96(3.63–4.31)	5.19(4.69–5.74)	
Hispanic	3.86(3.59–4.14)	3.47(3.17–3.80)	4.53(4.03–5.09)	
Non-Hispanic White	76.87(76.02–77.69)	79.60(78.61–80.56)	72.10(70.58–73.58)	
Non-Hispanic Black	9.15(8.72–9.60)	7.51(7.04–8.01)	12.00(11.18–12.87)	
Other race	5.72(5.22–6.27)	5.46(4.90–6.09)	6.17(5.24–7.26)	
Marital				<0.001
Married/living with a partner	62.53(61.29–63.76)	66.92(65.34–68.47)	54.88(52.89–56.85)	
Never married	6.33(5.74–6.98)	5.66(4.95–6.47)	7.50(6.51–8.62)	
Separated/divorced/widowed	31.14(29.99–32.31)	27.41(25.98–28.90)	37.62(35.76–39.52)	
Education				<0.001
< High school	17.66(16.86–18.49)	13.64(12.74–14.60)	24.67(23.19–26.21)	
High school	25.38(24.25–26.55)	23.54(22.11–25.03)	28.59(26.77–30.49)	
> High school	56.96(55.67–58.23)	62.82(61.20–64.42)	46.73(44.71–48.77)	
Income poverty ratio				<0.001
< 1.3	22.10(21.20–23.03)	15.20(14.26–16.19)	34.14(32.39–35.93)	
(1.3–3.5)	38.41(37.15–39.68)	36.36(34.76–37.99)	41.97(39.99–43.97)	
≥3.5	39.49(38.13–40.86)	48.44(46.70–50.19)	23.89(21.98–25.91)	
Smoke				<0.001
Yes	52.84(51.52–54.16)	48.72(46.99–50.44)	60.03(58.05–61.99)	
No	47.16(45.84–48.48)	51.28(49.56–53.01)	39.97(38.01–41.95)	
Alcohol use				<0.001
Yes	75.92(74.86–76.94)	77.19(75.83–78.49)	73.69(72.02–75.30)	
No	24.08(23.06–25.14)	22.81(21.51–24.17)	26.31(24.70–27.98)	

Among the 11,234 participants, 63.54% were non-frail, and 36.46% as frail. Stratifying by different levels of lnSIRI, significant differences in frailty, age, gender, race, education, marital status, income and poverty ratio, alcohol consumption, and smoking status were observed (*p* < 0.05). The proportions of the four categories of frailty were 34.96, 29.48, 34.43, and 46.69%, as illustrated in Table 2.

**Table 2:** Baseline characteristics of the study population stratified by quartiles of lnSIRI value.

	lnSIRI total	Q1(≥ − 0.29)	Q2(−0.29 to 0.11)	Q3(0.11–0.52)	Q4(≥0.52)	*p*-value
Sample size	11,234	2,808	2,808	2,808	2,810	
Year	64.75 ± 0.13	62.73 ± 0.25	63.94 ± 0.26	65.15 ± 0.26	66.77 ± 0.26	<0.001
Gender						<0.001
Male	45.95(44.63–47.27)	32.51(30.00–35.13)	41.42(38.78–44.11)	49.41(46.79–52.02)	57.71(55.21–60.17)	
Female	54.05(52.73–55.37)	67.49(64.87–70.00)	58.58(55.89–61.22)	50.59(47.98–53.21)	42.29(39.83–44.79)	
Race						<0.001
Mexican American	4.41(4.13–4.71)	5.52(4.83–6.30)	4.54(4.00–5.15)	4.32(3.81–4.90)	3.48(3.01–4.02)	
Hispanic	3.86(3.59–4.14)	4.63(4.04–5.29)	4.03(3.52–4.62)	3.59(3.09–4.17)	3.33(2.84–3.90)	
Non-Hispanic White	76.87(76.02–77.69)	62.44(60.09–64.73)	77.76(76.07–79.37)	80.43(78.86–81.91)	83.87(82.52–85.15)	
Non-Hispanic Black	9.15(8.72–9.60)	19.40(17.97–20.93)	8.27(7.49–9.13)	6.17(5.53–6.88)	4.86(4.28–5.50)	
Other race	5.72(5.22–6.27)	8.02(6.82–9.41)	5.39(4.42–6.55)	5.49(4.53–6.64)	4.46(3.69–5.39)	
Education						0.010
< High school	17.66(16.86–18.49)	19.21(17.53–21.02)	15.73(14.26–17.33)	17.49(15.96–19.13)	18.53(16.91–20.27)	
High school	25.38(24.25–26.55)	24.56(22.28–26.99)	25.10(22.83–27.52)	25.40(23.16–27.78)	26.30(24.16–28.55)	
> High school	56.96(55.67–58.23)	56.23(53.54–58.88)	59.16(56.57–61.71)	57.11(54.56–59.63)	55.18(52.70–57.62)	
Marital status						0.003
Married/living with a partner	62.53(61.29–63.76)	61.06(58.44–63.61)	63.50(60.98–65.95)	63.27(60.78–65.68)	62.00(59.61–64.33)	
Never married	6.33(5.74–6.98)	8.21(6.91–9.74)	5.53(4.53–6.72)	5.86(4.81–7.13)	6.11(4.94–7.55)	
Separated/divorced/widowed	31.14(29.99–32.31)	30.73(28.41–33.15)	30.98(28.67–33.39)	30.87(28.60–33.24)	31.89(29.73–34.12)	
Income poverty ratio						<0.001
< 1.3	22.10(21.20–23.03)	25.54(23.56–27.63)	20.46(18.72–22.31)	21.27(19.57–23.07)	21.85(20.11–23.70)	
(1.3–3.5)	38.41(37.15–39.68)	37.34(34.73–40.02)	35.97(33.51–38.51)	37.64(35.19–40.15)	42.47(40.03–44.94)	
≥3.5	39.49(38.13–40.86)	37.12(34.31–40.02)	43.57(40.83–46.35)	41.09(38.42–43.82)	35.68(33.15–38.29)	
Smoke						<0.001
Yes	52.84(51.52–54.16)	45.96(43.23–48.72)	49.30(46.62–51.99)	55.08(52.45–57.68)	59.60(57.11–62.05)	
No	47.16(45.84–48.48)	54.04(51.28–56.77)	50.70(48.01–53.38)	44.92(42.32–47.55)	40.40(37.95–42.89)	
Alcohol use						<0.001
Yes	75.92(74.86–76.94)	71.09(68.66–73.41)	76.16(74.03–78.16)	77.10(75.07–79.01)	78.32(76.34–80.18)	
No	24.08(23.06–25.14)	28.91(26.59–31.34)	23.84(21.84–25.97)	22.90(20.99–24.93)	21.68(19.82–23.66)	
Frailty						<0.001
No	63.54(62.3–64.76)	65.04(62.45–67.53)	70.52(68.13–72.80)	65.57(63.15–67.92)	53.31(50.82–55.79)	
Yes	36.46(35.24–37.7)	34.96(32.47–37.55)	29.48(27.20–31.87)	34.43(32.08–36.85)	46.69(44.21–49.18)	

Stratifying by different levels of lnSII, significant differences in frailty, race, education, marital status, income and poverty ratio, and smoking status were observed (*p* < 0.05). The proportions of the four frailty categories were 35.91, 31.12, 33.05, and 45.35%, as illustrated in Table 3.

**Table 3:** Baseline characteristics of the study population stratified by quartiles of lnSII value.

	lnSII total	Q1(≤5.78)	Q2(5.78–6.14)	Q3(6.14–6.5)	Q4(≥6.5)	*p*-value
Sample size	11,234	2,808	2,808	2,808	2,810	
Year	64.75 ± 0.13	64.54 ± 0.26	64.50 ± 0.26	64.91 ± 0.26	65.00 ± 0.26	0.170
Gender						0.924
Male	45.95(44.63–47.27)	46.39(43.64–49.15)	46.17(43.52–48.85)	45.80(43.18–48.44)	45.53(43.04–48.05)	
Female	54.05(52.73–55.37)	53.61(50.85–56.36)	53.83(51.15–56.48)	54.20(51.56–56.82)	54.47(51.95–56.96)	
Race						<0.001
Mexican American	4.41(4.13–4.71)	4.65(4.05–5.33)	4.83(4.25–5.49)	4.40(3.88–4.98)	3.81(3.31–4.37)	
Hispanic	3.86(3.59–4.14)	4.12(3.57–4.74)	4.32(3.76–4.95)	3.48(3.02–4.01)	3.57(3.06–4.18)	
Non-Hispanic White	76.87(76.02–77.69)	67.51(65.32–69.62)	76.17(74.46–77.81)	79.91(78.35–81.39)	82.10(80.65–83.47)	
Non-Hispanic Black	9.15(8.72–9.60)	16.92(15.62–18.31)	8.68(7.87–9.57)	6.89(6.19–7.67)	5.53(4.92–6.20)	
Other race	5.72(5.22–6.27)	6.80(5.57–8.28)	6.00(5.08–7.06)	5.32(4.40–6.41)	4.99(4.14–6.00)	
Education						0.013
< High school	17.66(16.86–18.49)	19.18(17.49–20.99)	16.47(14.96–18.10)	17.19(15.64–18.87)	18.03(16.47–19.70)	
High school	25.38(24.25–26.55)	25.49(23.14–28.00)	23.95(21.80–26.23)	26.88(24.55–29.35)	25.20(23.09–27.42)	
> High school	56.96(55.67–58.23)	55.33(52.62–58.01)	59.58(57.04–62.07)	55.92(53.33–58.49)	56.77(54.31–59.21)	
Marital status						<0.001
Married/living with a partner	62.53(61.29–63.76)	61.91(59.32–64.43)	66.82(64.41–69.15)	61.57(59.05–64.04)	59.87(57.42–62.27)	
Never married	6.33(5.74–6.98)	7.86(6.58–9.37)	4.98(4.02–6.15)	5.96(4.90–7.23)	6.75(5.55–8.19)	
Separated/divorced/widowed	31.14(29.99–32.31)	30.23(27.94–32.62)	28.20(26.02–30.49)	32.46(30.13–34.89)	33.38(31.14–35.69)	
Income poverty ratio						0.002
< 1.3	22.10(21.20–23.03)	24.25(22.32–26.30)	21.14(19.37–23.02)	20.52(18.84–22.30)	22.84(21.08–24.71)	
(1.3–3.5)	38.41(37.15–39.68)	37.69(35.09–40.36)	37.17(34.71–39.70)	39.01(36.51–41.57)	39.58(37.16–42.04)	
≥3.5	39.49(38.13–40.86)	38.06(35.24–40.96)	41.69(38.97–44.47)	40.47(37.79–43.21)	37.58(35.00–40.23)	
Smoke						<0.001
Yes	52.84(51.52–54.16)	50.00(47.24–52.76)	49.35(46.69–52.01)	53.61(50.97–56.24)	57.72(55.20–60.21)	
No	47.16(45.84–48.48)	50.00(47.24–52.76)	50.65(47.99–53.31)	46.39(43.76–49.03)	42.28(39.79–44.80)	
Alcohol use						0.311
Yes	75.92(74.86–76.94)	74.71(72.45–76.84)	75.63(73.43–77.71)	76.27(74.17–78.24)	76.82(74.83–78.69)	
No	24.08(23.06–25.14)	25.29(23.16–27.55)	24.37(22.29–26.57)	23.73(21.76–25.83)	23.18(21.31–25.17)	
Frailty						<0.001
No	63.54(62.3–64.76)	64.09(61.44–66.65)	68.88(66.49–71.18)	66.95(64.56–69.27)	54.65(52.15–57.13)	
Yes	36.46(35.24–37.7)	35.91(33.35–38.56)	31.12(28.82–33.51)	33.05(30.73–35.44)	45.35(42.87–47.85)	

### 3.2 The relationship between lnSII, lnSIRI and frailty

Three weighted logistic regression models were employed to investigate the association between lnSII, lnSIRI, and frailty. The odds ratios (OR) and 95% confidence intervals (CI) for the ratio of frailty’s correlation with lnSII and lnSIRI are presented in Table 4. Model 1, unadjusted; Model 2, adjusted for age, gender, race, education, marital status, income-to-poverty ratio; Model 3, additional adjustment for smoking and drinking status based on Model 2. There is a consistently significant positive correlation between lnSII, lnSIRI, and frailty in all three models. The impact of lnSII on frailty was as follows: Model 1 (OR = 1.34, 95% CI: 1.21–1.48), Model 2 (OR = 1.41, 95% CI: 1.26–1.57), Model 3 (OR = 1.38, 95% CI: 1.24–1.54). When assessing different levels of lnSII, compared to the second quartile of lnSII, the first and third quartiles did not show a significant difference in the probability of frailty. At the same time, the likelihood significantly increased in the fourth quartile: Model 1 (OR = 1.84, 95% CI: 1.58–2.13), Model 2 (OR = 1.86, 95% CI: 1.59–2.17), Model 3 (OR = 1.82, 95% CI: 1.55–2.12). Furthermore, the trend *p*-values for all three models were below 0.001.

**Table 4:** Association between lnSII, lnSIRI and frailty.

	Model 1 OR (95% CI)	*p*-value	Model 2 OR (95% CI)	*p*-value	Model 3 OR (95% CI)	*p*-value
lnSII	1.34(1.21–1.48)	<0.0001	1.41(1.26–1.57)	<0.0001	1.38(1.24–1.54)	<0.0001
lnSII quartiles						
Q1(≤5.78)	1.24(1.06–1.45)	0.0073	1.16(0.98–1.36)	0.0787	1.16(0.99–1.37)	0.071
Q2(5.78–6.14)	Reference	Reference	Reference	Reference	Reference	Reference
Q3(6.14–6.5)	1.09(0.94–1.27)	0.2562	1.09(0.93–1.27)	0.309	1.07(0.92–1.26)	0.3854
Q4(≥6.5)	1.84(1.58–2.13)	<0.0001	1.86(1.59–2.17)	<0.0001	1.82(1.55–2.12)	<0.0001
*p* trend	<0.001		<0.001		<0.001	
lnSIRI	1.45(1.32–1.58)	<0.0001	1.73(1.56–1.91)	<0.0001	1.69(1.53–1.88)	<0.0001
lnSIRI quartiles						
Q1(≥ − 0.29)	1.29(1.10–1.51)	0.0019	1.09(0.93–1.29)	0.2897	1.10(0.94–1.30)	0.2367
Q2(−0.29 to 0.11)	Reference	Reference	Reference	Reference	Reference	Reference
Q3(0.11–0.52)	1.26(1.08–1.47)	0.0038	1.32(1.12–1.55)	0.0008	1.30(1.10–1.53)	0.0016
Q4(≥0.52)	2.09(1.80–2.44)	<0.0001	2.34(1.99–2.76)	<0.0001	2.29(1.95–2.70)	<0.0001
*p* trend	<0.001		<0.001		<0.001	

The impact of lnSIRI on frailty was as follows: Model 1 (OR = 1.45, 95% CI: 1.32–1.58), Model 2 (OR = 1.73, 95% CI: 1.56–1.91), and Model 3 (OR = 1.69, 95% CI: 1.53–1.88). When assessing different levels of lnSIRI, compared to the second quartile of lnSIRI, the first quartile did not show a significant difference in the probability of frailty. In contrast, the probabilities significantly increased in the third and fourth quartiles. For the third quartile: Model 1 (OR = 1.26, 95% CI: 1.08–1.47), Model 2 (OR = 1.32, 95% CI: 1.12–1.55), Model 3 (OR = 1.30, 95% CI: 1.10–1.53); For the fourth quartile: Model 1 (OR = 2.09, 95% CI: 1.80–2.44), Model 2 (OR = 2.34, 95% CI: 1.99–2.76), Model 3 (OR = 2.29, 95% CI: 1.95–2.70). Furthermore, the trend *p*-values for all three models were below 0.001.

**Figure 2:**
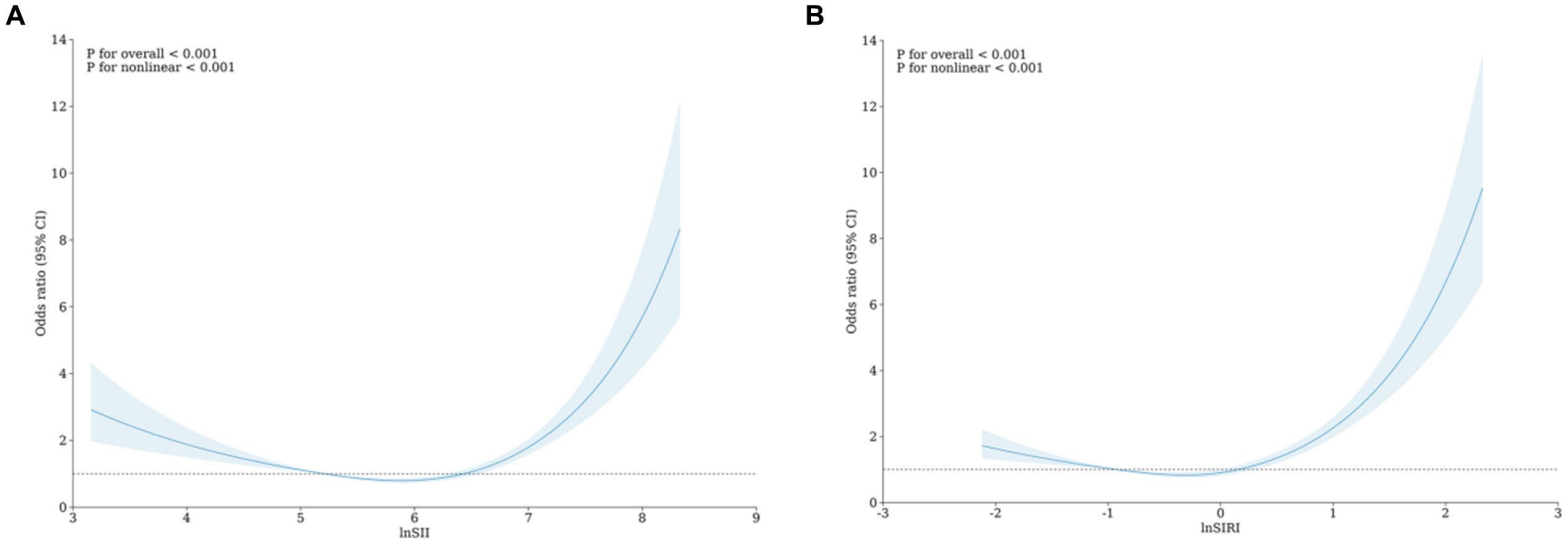
RCS analysis of lnSII **(A)** and lnSIRI **(B)** with the odds ratio of frailty.

We also used RCS to visualize the association between lnSII, lnSIRI, and frailty. After adjusting for all covariates in the primary analysis Model 3 mentioned above, we observed a non-linear correlation between lnSII, lnSIRI, and frailty (Figure 2).

### 3.3 Stratified analyses and interaction test

**Figure 3:**
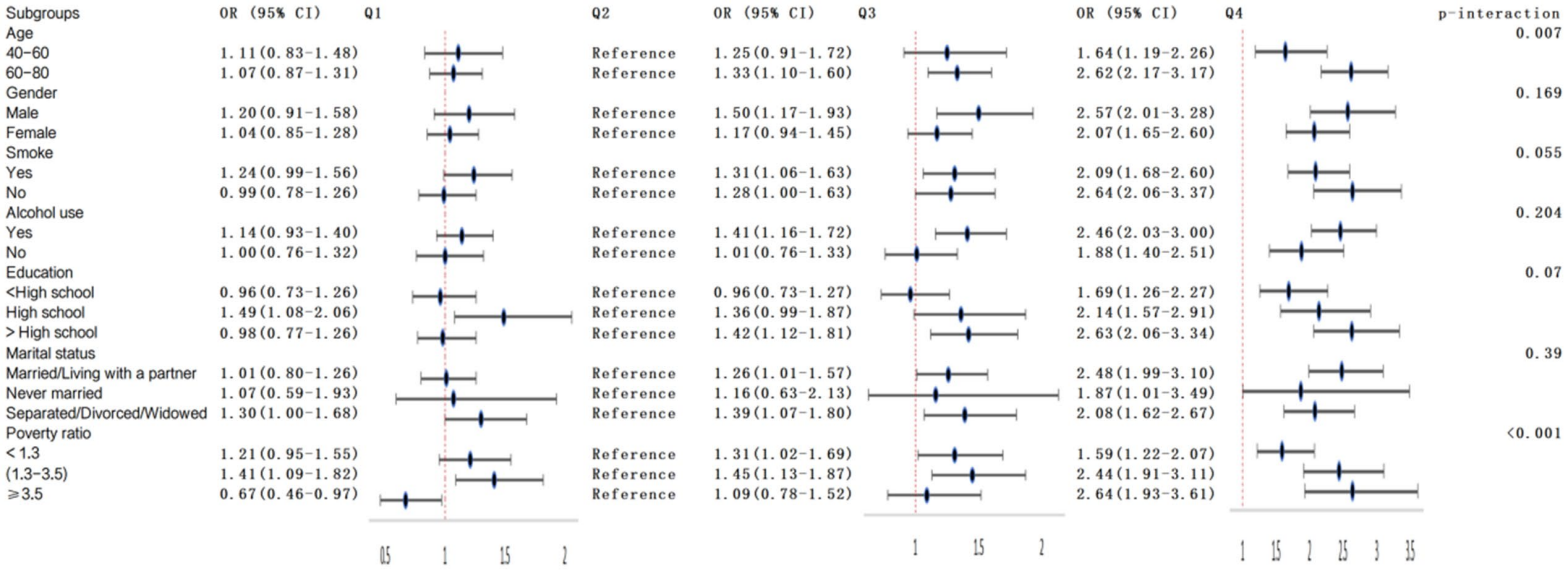
Subgroup analysis and interaction of the association between lnSIRI and frailty.

The interaction tests in the subgroup analysis revealed that the impact of lnSIRI on frailty varied with age (*p* for interaction = 0.007) and income-to-poverty ratio (*p* for interaction <0.001). However, gender, race, education, marital status, smoking, and alcohol consumption did not influence this positive correlation (*p* for interaction >0.05), as illustrated in Figure 3.

**Figure 4:**
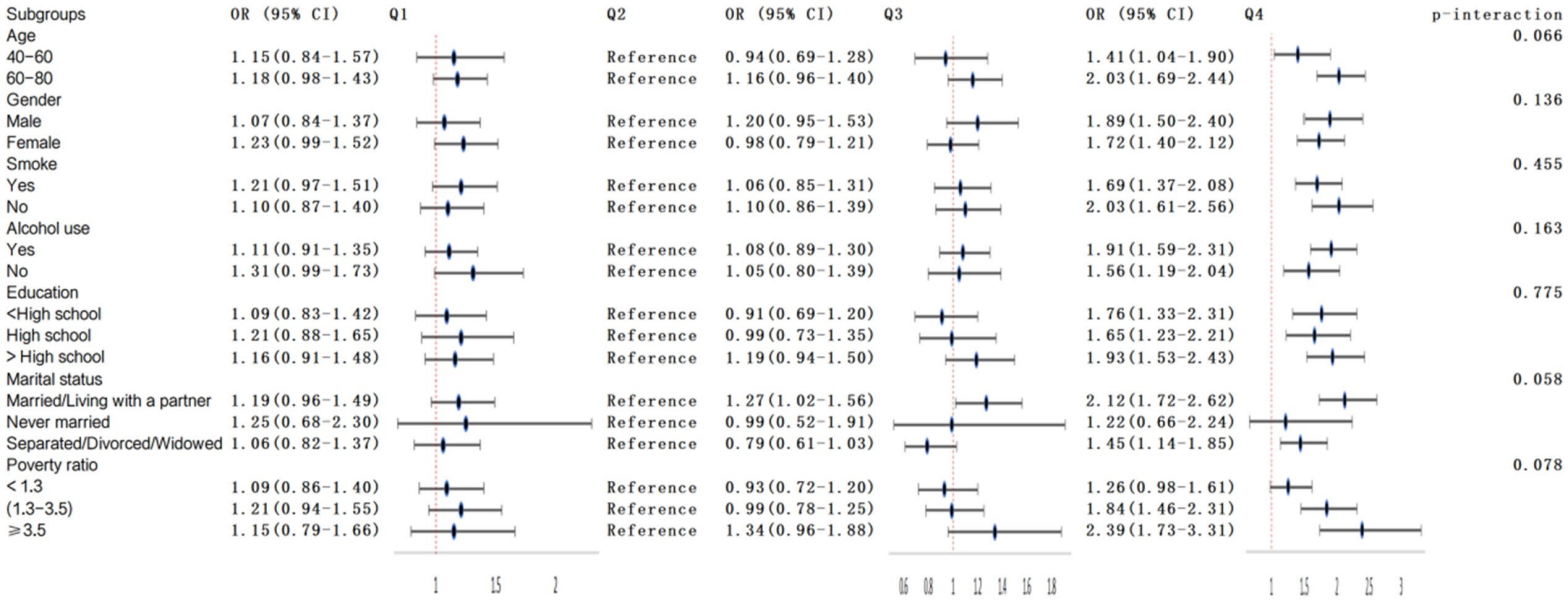
Subgroup analysis and interaction of the association between lnSII and frailty.

The relationship between lnSII and frailty showed no statistically significant differences across different strata, indicating that age, gender, race, education, marital status, income-to-poverty ratio, smoking, and alcohol consumption did not significantly affect this positive correlation (*p* for interaction >0.05), as depicted in Figure 4.

## 4 Discussion

This cross-sectional study investigated the relationship between lnSII, lnSIRI, and frailty using weighted logistic regression. We included 11,234 adults aged 40 years and older. The study’s results indicated a positive correlation between lnSII and lnSIRI with frailty. This finding aligns with previous research, which suggested that individuals with higher neutrophil-to-lymphocyte ratio and higher SII levels have an increased risk of frailty events (17). Furthermore, our study revealed that age and income poverty ratio influence the association between lnSIRI and frailty. In contrast, the association between lnSII and frailty is relatively unaffected by other factors. We also observed several differences between frail and non-frail individuals. Frail individuals were more likely to be female, have lower educational attainment, lower income poverty ratio, less likely to be married/cohabiting, smokers, and had higher levels of lnSII and lnSIRI. Previous studies have also shown that the frail group is more likely to be female, have lower educational attainment, and have lower income (31, 32).

Research indicates that the prevalence of frailty among older adults is between 12 and 24% in 62 countries and regions (33). Frailty has been shown to have varying degrees of impact on the prognosis of older adults with cardiovascular diseases, increasing the incidence and mortality rates of cardiovascular disease patients (34). It becomes an independent risk factor for various major adverse cardiovascular events, including death, stroke, readmission for heart failure, and postoperative cardiac complications (35). Additionally, frail patients are at a higher risk of developing sepsis, pneumonia, and kidney failure compared to non-frail individuals (36). However, the pathological mechanisms leading to frailty remain unclear, and there is a lack of recognized, accurate, and reliable biological markers for frailty (37–39). Future research efforts should focus on understanding the pathogenesis of frailty to improve early diagnosis and intervention, thereby alleviating the burden on global healthcare systems.

The study points out that elevated levels of inflammatory markers are commonly found in older adults, and inflammation may be a primary factor leading to frailty (40, 41). The association between changes in the immune system and frailty involves multiple pathways, with neutrophils being a crucial biomarker for innate immunity, platelets potentially contributing to immune function, and lymphocytes providing rich information about adaptive immunity (42, 43). SII and SIRI have demonstrated exceptional validity as emerging biomarkers in various diseases (44). SII’s predictive ability for major cardiovascular events in coronary heart disease patients undergoing coronary intervention surpasses traditional risk factors (45). Both SII and SIRI are closely correlated with cardiovascular and all-cause mortality. These studies emphasize the role of managing inflammatory markers in frailty among middle-aged and older adults and suggest that emerging biomarkers like SII and SIRI could be powerful tools for assessing and managing the health of middle-aged and older individuals (46, 47).

The findings of this study reveal a positive correlation between SII and SIRI with frailty, especially with SIRI exhibiting a more significant association. The study holds general significance due to its large sample size and representative sample selection. However, it is essential to note some limitations. Firstly, the study adopts a cross-sectional design, thus preventing the establishment of a causal relationship between SII, SIRI, and frailty. Secondly, some unaccounted confounding factors could impact the accurate assessment of the genuine associations.

## 5 Conclusion

This cross-sectional study provides compelling evidence indicating a positive correlation between systemic inflammatory biomarkers (SIRI and SII) and frailty. Given the ease of assessment of SIRI and SII in the laboratory, they can serve as cost-effective predictive factors for future frailty occurrences. This offers feasibility and guiding information for further interventions targeting the immune system to reduce the incidence of frailty.

## Data availability statement

The original contributions presented in the study are included in the article/Supplementary material, further inquiries can be directed to the corresponding author.

## Author contributions

HZ: Conceptualization, Data curation, Methodology, Software, Visualization, Writing – original draft. XL: Supervision, Writing – review & editing. XW: Data curation, Visualization, Writing – original draft. YJ: Conceptualization, Supervision, Validation, Visualization, Writing – review & editing.

## Footnotes

^1^https://www.cdc.gov/nchs/nhanes/index.htm
